# Do in-person and computer-based brief alcohol interventions reduce tobacco smoking among general hospital patients? Secondary outcomes from a randomized controlled trial

**DOI:** 10.1186/s13722-023-00425-7

**Published:** 2023-11-13

**Authors:** Filipa Krolo-Wicovsky, Sophie Baumann, Anika Tiede, Gallus Bischof, Ulrich John, Beate Gaertner, Jennis Freyer-Adam

**Affiliations:** 1 Institute for Medical Psychology, University Medicine Greifswald, Walther-Rathenau-Str. 48, 17475 Greifswald, Germany; 2 German Centre for Cardiovascular Research, Partner site Greifswald, Fleischmannstr. 42-44, 17475 Greifswald, Germany; 3 Department of Methods in Community Medicine, Institute of Community Medicine, University Medicine Greifswald, Walther-Rathenau-Str. 48, 17475 Greifswald, Germany; 4 Department of Psychiatry and Psychotherapy, University of Lübeck, Ratzeburger Allee 160, 23538 Lübeck, Germany; 5 Department of Prevention Research and Social Medicine, Institute of Community Medicine, University Medicine Greifswald, Greifswald, Germany; 6 Department of Epidemiology and Health Monitoring, Robert Koch Institute Berlin, Berlin, General-Pape-Str. 62-66, 12101 Berlin, Germany

**Keywords:** Brief intervention, Alcohol, Tobacco, Smoking, Spill-over, Computer invention, Counselling

## Abstract

**Background:**

At-risk alcohol use and tobacco smoking often co-occur. We investigated whether brief alcohol interventions (BAIs) among general hospital patients with at-risk alcohol use may also reduce tobacco smoking over 2 years. We also investigated whether such effects vary by delivery mode; i.e. in-person versus computer-based BAI.

**Methods:**

A proactively recruited sample of 961 general hospital patients with at-risk alcohol use aged 18 to 64 years was allocated to three BAI study groups: in-person BAI, computer-based BAI, and assessment only. In-person- and computer-based BAI included motivation-enhancing intervention contacts to reduce alcohol use at baseline and 1 and 3 months later. Follow-ups were conducted after 6, 12, 18 and 24 months. A two-part latent growth model, with self-reported smoking status (current smoking: yes/no) and number of cigarettes in smoking participants as outcomes, was estimated.

**Results:**

Smoking participants in computer-based BAI smoked fewer cigarettes per day than those assigned to assessment only at month 6 (mean_net change_ = − 0.02; 95% confidence interval = − 0.08–0.00). After 2 years, neither in-person- nor computer-based BAI significantly changed smoking status or number of cigarettes per day in comparison to assessment only or to each other (*p*s ≥ 0.23).

**Conclusions:**

While computer-based BAI also resulted in short-term reductions of number of cigarettes in smoking participants, none of the two BAIs were sufficient to evoke spill-over effects on tobacco smoking over 2 years. For long-term smoking cessation effects, multibehavioural interventions simultaneously targeting tobacco smoking along with at-risk alcohol use may be more effective.

*Trial registration number*: NCT01291693.

## Background

Alcohol drinking and tobacco smoking can have cumulative adverse effects on health [1, 2]. This has been revealed by data, particularly for upper aerodigestive health disorders [3]. In Europe, the prevalence of daily tobacco and alcohol consumption is still among the highest in the world [2]. In a general population sample in Germany, the proportion of daily and occasional smoking persons among adult at-risk alcohol drinking persons was 55.1%, and the proportion of at-risk drinking persons among adult daily and occasional smoking persons was 30.0% [4]. Consumption of alcohol may stimulate or support tobacco smoking and vice versa [5, 6]; and quitting one substance use may result in reduction of the other [7, 8]. Evidence from different fields including epidemiological, clinical, psychological, and genetic studies makes this likely [9–11]. For example, ethanol and nicotine act on the same neural pathways and in the same areas in the brain [1, 12]. Both substances are often consumed with similar motivation, such as emotion regulation (i.e. enhancing euphoric feelings or reducing tension) [13] or through psychosocial influences by family or peers (i.e. model learning and sense of belonging when also consuming) [14].

Given this evidence, brief alcohol interventions (BAIs) to stop or reduce alcohol risk drinking might have the potential to reduce tobacco smoking, even if not primarily aimed at. BAIs have been shown to reduce alcohol use in health care populations [15, 16]. However, little is known about their effects on tobacco smoking. Only few studies have investigated such spill-over effects of BAIs on tobacco smoking. Two meta-analyses that identified 7 studies in health care settings and 6 studies among adolescents and young adults, respectively, found no positive effect of BAIs on tobacco smoking [17, 18]. A recent alcohol intervention conducted among adolescents successfully reduced chewing tobacco, marijuana and subscripted drugs use, but not cigarettes per day [19]. Findings are mostly limited to adolescents and young adults and to effects up to month 12 after intervention. This short follow-up time is a main limitation since brief smoking cessation and alcohol interventions revealed that intervention effects increased beyond year 1 in health care patients [20–22]. Longer term follow-ups might be needed to observe such increasing behavioural and health effects from motivation-enhancing interventions [20–23]. The analysis of long-term outcome data beyond one year of follow-up, especially on tobacco use as recommended for studies on brief alcohol interventions [18, 19].

Most of the BAIs investigated with regards to spill-over effects were delivered in-person [17, 18]. Electronic BAIs have been suggested and proven to be effective in reducing self-reported alcohol use: For instance, two reviews [24, 25] including 42 and 57 studies conducted in primary care and the general population, respectively, found that electronic BAIs were effective in reducing alcohol use or harm in participants. For example, one of the reviews reported an average reduction of up to three standard drinks per week in the intervention group compared to the control group [25]. However, evidence on the comparative efficacy of different intervention delivery channels, such as in-person versus computer-based BAIs, is still scarce, especially concerning spill-over effects among adults in health care.

The two aims of this study were, first, to investigate whether computer-based and in-person delivered BAIs may reduce tobacco smoking over two years and, second, whether such an effect differs by delivery mode, that is whether in-person or computer-based BAIs are more or less effective in also reducing tobacco smoking.

## Methods

Data were derived from a three-arm randomized controlled trial ‘Testing delivery channels of individualized motivationally tailored alcohol interventions among general hospital patients: in-person versus computer-based, PECO’ [20, 26]. PECO is registered at ClinicalTrials.gov (NCT01291693). The ethics committee of the University Medicine Greifswald approved the study prior to data collection (BB07/10 and BB105/13). All trial participants provided informed written consent.

### Participants

In 2011–2012, participants were recruited on 13 wards in four medical departments at the University Medicine Hospital Greifswald in Germany: internal medicine, general surgery, trauma surgery and ear-nose-throat. Intensive care units were excluded. All consecutively admitted patients aged 18–64 years, were approached by a research assistant and asked to respond to an electronic self-administrative lifestyle screening. Patients cognitively or physically incapable or terminally ill, with highly infectious diseases, discharged or transferred outside the study area within the first 24 h, already recruited previously, with insufficient German language skills, or employed at the conducting research institute were excluded. Those study participants who screened positive for at-risk alcohol use, i.e. exceeding values of ≥ 4 for women and ≥ 5 for men on the Alcohol Use Disorder Identification Test (AUDIT)-Consumption [28, 29], were eligible for trial inclusion. These values correspond to German recommendations suggesting to drink no more than 12 g/ 14 g of pure alcohol per day and no more than 3/4 drinks per occasion for women/ men, respectively [30]. Patients with a total AUDIT score ≥ 20 [31, 32] were excluded due to insufficient BAI efficacy in people with more severe alcohol problems [33]. Patients with no telephone were also excluded.

**Fig. 1:**
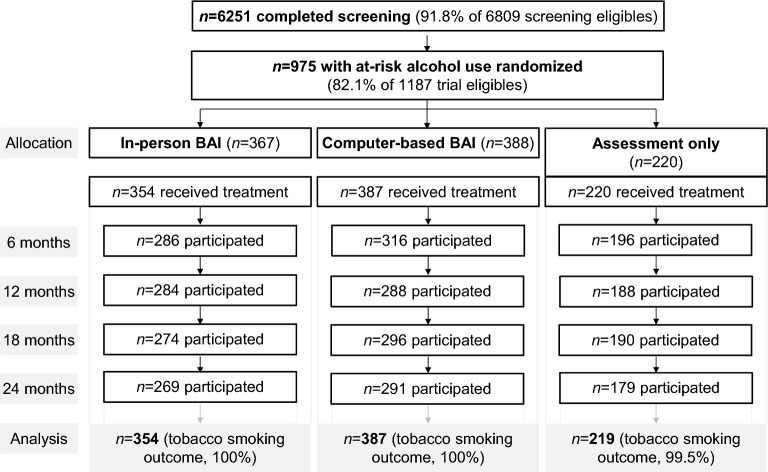
Participant flow stratified by study group

Among 10,593 patients aged 18–64 years who had been admitted to the participating wards, 6809 were eligible for screening, and 6251 (92%) completed the screening. Of the 1,187 patients eligible for trial participation, 961 (81%) received their allocated intervention. One participant with missing baseline covariate data was excluded from analysis. The final sample for analysis included 960 patients with at-risk alcohol use (Fig. 1). Participant flow according to CONSORT is reported in more detail elsewhere [20].

### Brief alcohol interventions

As described elsewhere [20, 26], in-person BAI and computer-based BAI were individually tailored based on psychological behaviour change theory and included three intervention contacts (i.e. baseline, 1 and 3 months after baseline), normative feedback (i.e. on own data in comparison to others) and ipsative feedback (i.e. on own changes over time). Prior to each intervention, current alcohol use and the four dimensions of the transtheoretical model of behaviour change [34] (i.e. stage of change, processes of change, decisional balance, self-efficacy) in terms of alcohol use were assessed using tablet computers at baseline and telephone interviews 1 and 3 months after baseline. Interventions are expected to be most effective when they are individually tailored to the person's current motivational stage of change (precontemplation, contemplation, preparation, action, maintenance) [34], and when all dimensions of underlying behaviour-change models are used [35, 36].

In-person BAI and computer-based BAI were delivered face-to-face on the ward (i.e. at baseline), and by phone/ mail (i.e. 1 and 3 months after baseline). In-person BAI was primarily delivered by psychologists trained in motivational interviewing [37] based behaviour change counselling. Among all participants, 83% received two or three consultations. Across all three consultations, in-person BAI participants received 35 min of counselling (median) concerning their alcohol use. Audiotaped in-person BAI sessions coded with global ratings of the motivational interviewing treatment integrity code showed that in-person BAI was delivered with acceptable adherence to motivational interviewing [20, 38]. Computer-based BAI consisted of tailored 3–4-page computer-generated feedback letters, written in a patient-accepting, supportive and non-confrontational style. In addition, they received stage-matched manuals. Of the participants, 89% received two or three feedback letters. Assessment only received minimal assessment at baseline only and they were not contacted 1 and 3 months after baseline.

An allocation ratio of 2 (in-person BAI): 2 (computer-based BAI): 1 (assessment only) was used. To prevent study groups from exchanging intervention information, study group allocation rotated weekly over wards.

### Follow-up

Follow-up data were collected 6, 12, 18 and 24 months after recruitment using primarily computer-assisted telephone interviews (88%) and face-to-face interviews. Incentives consisting of vouchers for purchases of daily needs were sent out to participants before the follow-up at month 12 (5€) and after follow-up participation at months 6 (10€), 18 (15€), and 24 (20€). Overall, follow-up participation per time-point was 77–83%. The interviewers were not informed about group allocation. Of all computer-assisted telephone interviews, 64% were conducted by student interviewers (97/47/49/60% at months 6/12/18/24) and 36% by research assistants who were involved in recruitment 1–2 years earlier.

### Measures

#### Outcome

In our study, we included the following two indicators for tobacco smoking: [1]. Smoking status was assessed by the question “Are you a smoker currently?” including four response categories: “No, I have never smoked”, “No, I don’t smoke anymore”, “Yes, I smoke daily” and “Yes, I smoke occasionally”. For the purpose of this study, participants who reported current daily and occasional smoking were defined as current smoking participants, all others (never and former daily) as current non-smoking participants [2]. Number of cigarettes per day was assessed among current smoking participants, by asking: “How many cigarettes/cigarillos/pipes/cigars do you usually consume on a day, when you smoke?”. Given the small proportion of smoking participants using cigarillos, pipes and cigars (5.8% at baseline), we did not differentiate among tobacco products. Current non-smoking participants were deemed as equivalent to 0 cigarettes per day. Both outcomes on tobacco smoking were specified before carrying out the statistical analysis.

#### Covariates

Sociodemographic data included sex (male/female), age (in years), living in a partnership or being married (no/yes), employment status (employed/ unemployed/ other, e.g., pupil, student, or retiree) and level of school education (low/ medium/ high). The hospital department to which the patients were admitted (internal medicine, surgical medicine, trauma surgery, ear-nose-throat) was recorded. Self-rated health was assessed using a single item “Would you say your health in general is excellent, very good, good, fair, poor?” [39]. Anxiety and depressive symptoms were assessed using the five-item mental health inventory [40, 41]. The score range was transformed to 0–100, with higher scores indicating better mental health. Alcohol use disorder symptoms were assessed using the AUDIT-C score (range: 0–12, mean centred) [29]. Number of alcohol use disorder symptoms (range: 0–7) was assessed as well, by coding each of the AUDIT items 4–10 as 1 if the respective symptom had been experienced in the past 12 months. The stage in the intention to change alcohol drinking (precontemplation, contemplation, preparation, action) was assessed by a 4-item staging algorithm, an adaption of measures previously introduced [42, 43].

#### Data analysis

To investigate the effects of in-person BAI and computer-based BAI on tobacco smoking over 24 months with five time points (baseline, 6, 12, 18 and 24 months), a two-part latent growth curve model [44] was used. Latent growth models allow to model complex nonlinear growth curves over time, to reflect variance in growth curves and to properly handle incomplete data [45]. In a latent growth model, repeated measures of the outcome are treated as indicators of latent growth variables that represent the outcome growth trajectory. Functional form and variability of the growth curves were tested via likelihood ratio tests. A two-part model was chosen due to high numbers of zeros in cigarettes per day, as 47.1% of all participants did not smoke currently. In the analysis, a binary model part (i.e. current smoking yes vs. no) and a continuous model part (i.e. number of cigarettes, if any) were created from cigarettes per day. Differences in change between the study groups were calculated as net changes from baseline to follow-up.

To measure in-person BAI and computer-based BAI effects on smoking status (binary model part), odds ratios (OR) and 95% confidence intervals (CI) were obtained. To measure in-person BAI and computer-based BAI effects on the number of cigarettes per day (continuous model part), squared estimates of mean net changes were obtained. Primary time point was month 24 and statistical significance was tested with p < 0.05. Analyses were adjusted for all baseline covariates and conducted using MPlus version 8.4 [46]. A maximum likelihood estimator with robust standard errors using numerical integration was chosen. Models were estimated under a missing at random assumption [47] using all available data. Multivariate logistic regression analyses including all covariates reported above revealed that dropout was significantly predicted by young age and hospitalization on surgical versus internal medicine wards (*p*s ≤ 0.02), but no other of the above reported variables. Also, there was no difference in the reported number of cigarettes per day across groups (*p* = 0.41).

## Results

### Sample characteristics

As described in more detail elsewhere [20], 718 (74.9%) of all the participants were male. The mean age was 40.9 years (standard deviation [SD] = 14.1) and 654 (68.1%) participants were in a partnership. A total of 190 (19.8%) had low, 533 (55.5%) medium, and 237 (24.7%) had high level of school. Among participants, 626 (65.2%) were employed and 393 (40.9%) had no intention to reduce or quit alcohol drinking (precontemplation stage). The mean AUDIT-C score was 6.0 (SD = 1.6) and the mean number of alcohol use disorder symptoms was 1.2 (SD = 1.5). Among participants, 508 (52.9%) smoked tobacco currently, with 417 (43.5%) doing so on a daily basis and 90 (9.4%) at least occasionally. The average number of cigarettes per day among daily and occasionally smoking participants was 14.0 (SD = 9.3), which is comparable to the amount smoked by smoking persons among the general population in Germany [48]. Observed information on tobacco smoking, stratified by study group are given for each time-point in Table 1. The outcome analysis, however, included all participants, regardless of follow-up participation.

**Table 1:** Observed values on both outcome measures

*Current tobacco smoking (n, %)*	Baseline	Month 6	Month 12	Month 18	Month 24
	*n* = 960	*n* = 751	*n* = 756	*n* = 759	*n* = 737
In-person BAI	188 (53.1)	125 (47.0)	141 (49.7)	137 (50.0)	140 (52.0)
Computer-based BAI	208 (53.8)	146 (49.8)	142 (49.7)	151 (51.0)	145 (50.0)
Assessment only	112 (51.1)	78 (40.6)	84 (45.2)	81 (42.9)	81 (45.5)

*Cigarettes per day among smoking participants (M, SD)*	n = 508	n = 348	n = 365	n = 364	n = 364
In-person BAI	14.1 (8.7)	13.2 (7.5)	14.0 (8.1)	14. 0 (7.1)	14.0 (8.3)
Computer-based BAI	14.1 (9.3)	12.5 (8.9)	12.6 (8.8)	12.9 (9.2)	13.2 (8.8)
Assessment only	13.8 (10.0)	13.1 (8.6)	12.7 (8.2)	13.5 (7.6)	13.1 (8.3)

*n* = number of cases; BAI = Brief alcohol intervention; *M* = mean; *SD* = standard deviation

### Smoking status over time with and without BAI

The binary model part revealed that none of the three study groups changed their smoking status significantly at any of the time points (month 24: *p*s ≥ 0.28), with no significant differences between any of the study groups (*p*s ≥ 0.23, Table 2).

**Table 2:** Study group differences in net changes in smoking status and cigarettes per day in smoking participants

Comparisons	Month 6		Month 12		Month 18		Month 24		
*Binary part (current smoking* = *1)*	*OR*	95% CI	*OR*	95% CI	*OR*	95% CI	*OR*	95% CI	*p*
In-person BAI vs. assessment only	1.07	0.89; 1.12	1.13	0.86; 1.48	1.18	0.88; 1.58	1.22	0.88; 1.69	0.23
Computer-based BAI vs. assessment only	1.12	0.93; 1.34	1.18	0.90; 1.54	1.17	0.87; 1.57	1.09	0.79; 1.51	0.57
In-person BAI vs. computer-based BAI	0.96	0.82; 1.12	0.96	0.76; 1.22	1.01	0.78; 1.31	1.12	0.84; 1.50	0.50

*Continuous part (cigarettes per day)*	Δ*M*	95% CI	Δ*M*	95% CI	Δ*M*	95% CI	Δ*M*	95% CI	*p*
In-person BAI vs. assessment only	0.00	− 0.04–0.00	− 0.01	− 0.08–0.01	0.00	− 0.07–0.02	0.00	− 0.04–0.08	0.92
Computer-based BAI vs. assessment only	− 0.02	− 0.08–0.00003	− 0.04	− 0.15–0.00	− 0.03	− 0.14–0.00	0.00	− 0.07–0.02	0.63
In-person BAI vs. computer-based BAI	0.01	0.00–0.04	− 0.01	0.01–0.09	0.01	− 0.01–0.10	0.00	− 0.02–0.08	0.58

Adjusted model. *OR* odds ratio of changing smoking status from smoking to non-smoking, *ΔM* differences in mean net change in cigarettes per day in smoking participants, *CI* confidence interval, *p* p-value

### Cigarettes per day over time with and without BAI

**Fig. 2:**
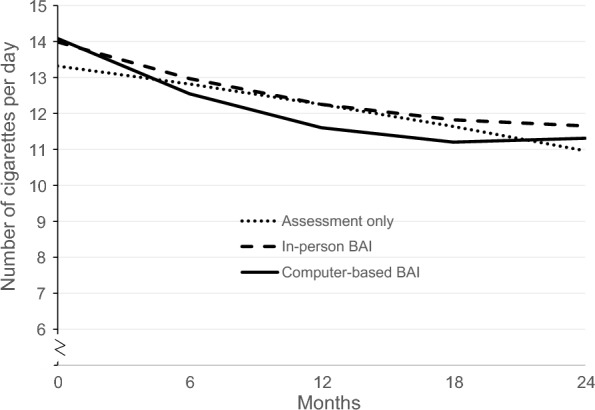
Estimated changes of number of cigarettes per day in smoking participants over 24 months

The continuous model part revealed that 24 months after receiving in-person BAI or computer-based BAI or assessment only, current smoking participants reduced the number of cigarettes per day significantly *within* all three study groups (*p*s ≤ 0.02). Reductions of cigarettes were observed at months 6 through 24 after computer-based BAI (Δ*M* − 0.04, 95% CI − 0.15; 0.00), at months 18 through 24 after in-person BAI (Δ*M* -0.08, 95% CI − 0.32; 0.00), and at month 24 after assessment only (Δ*M* − 0.11, 95% CI − 0.38; 0.00, Fig. 2).

Regarding differences between the intervention groups compared to assessment only, smoking participants who received the computer-based BAI smoked fewer cigarettes per day at month 6 (Δ*M* = − 0.02; 95% CI − 0.08; 0.00003, Table 2). However, at month 24, differences were not statistically significant (Δ*M* 0.00, 95% CI − 0.06; 0.01, *p* = 0.63). No significant differences were observed between in-person BAI and assessment only and between in-person BAI and computer-based BAI at month 24.

## Discussion

This study explored potential spill-over effects of computer-based and in-person delivered BAI on tobacco smoking in proactively recruited general hospital patients. Two main findings of this study are: First, within the first year, a computer-based intervention targeting alcohol use in at-risk alcohol users also decreased cigarettes per day among those who also smoked tobacco. Second, spill-over effects were no longer found two years later.

In line with previous systematic reviews [17, 18], the in-person BAI did not result in spill-over effects. However, the computer-based BAI did result in reduced smoking among those also smoking tobacco (52.9% of the sample) within the first year after intervention. Until now, little was known about such findings in the context of computer-based BAIs. When looking at effects of eHealth interventions targeting smoking only, we found large heterogeneity in outcome measures, making it difficult to compare our results. However, two studies also reported differences in cigarettes per day between study conditions: While in our study, the mean difference in change of cigarettes per day between computer-based BAI and assessment only was ΔM = − 0.02, the mean difference of the two studies directly targeting smoking was stronger, ranging between – 1.22 and – 5.1 [49, 50]. These findings seem plausible given the fact that our intervention initially targeted alcohol use. There are several potential explanations for why the computer-based BAI was nonetheless able to evoke tobacco smoking reduction effects within the first year after intervention. First, our target group included adults, while most studies on spill-over effects were conducted among adolescents and young adults. Conducting the study among a different target group might have led to the discovery of results, which were not found previously. Second, in comparison to one meta-analysis among adults that showed no spill-over effects [17], our data was collected more recently, i.e. after recorded changes of smoking behaviour in the general population, e.g. resulting from increased governmental tobacco cessation policies [51]. These may have encouraged reduced cigarette consumption in the population including our sample. Third, according to previous research, it may be expected that decreasing the use of one drug might decrease the use of another drug [7, 8]. As the computer-based intervention succeeded in reducing alcohol use over two years [20], it appears plausible that participants may also have reduced their smoking quantity either following or parallel to the reduction of alcohol use. Fourth, the subjective representation of success in having reduced alcohol use may support self-efficacy to reduce tobacco smoking as well [52]. This spill-over effect of the intervention might be a core advantage that has received too little attention in research about health behaviour change in the past. Fifth, the delivery mode of the intervention may have played a crucial role. Previous studies which showed no spill-over effects investigated in-person interventions only [17–19]. We found spill-over effects after computer-based intervention only. Possible explanations being that a) proactively recruited participants may have felt less problematized or stigmatized just by the fact that intervention was delivered by a rather unbinding feedback letter and not by a person, b) individualized feedback letters may have been (repeatedly) read whenever decided upon [53], and c) the core ideas of the transtheoretical model of behaviour change [34] may have been implemented more reliably in written feedback than in-person.

However, our expectation that such effects may also be observable in the long-term, given improved health-related outcomes following both BAIs investigated [22, 23], was not supported. One possible explanation could be that BAIs are not sufficient to evoke measurable long-term effects on tobacco smoking [18]. Some researchers suggest that tobacco smoking should be handled differently from other secondary substances in BAIs, as smoking has often been viewed as a behaviour difficult to change, even when directly targeted [18, 54].

For long-term cessation effects in at-risk alcohol drinking smoking persons, it may be crucial to target tobacco smoking at least simultaneously with at-risk alcohol use, when both substances co-occur. Feasibility and acceptability of such combined approaches among smoking and alcohol consuming individuals were found in one study [55]. Hence, multiple behaviour-change intervention approaches may be more promising, as also found by a systematic review of multi-behavioural interventions in adolescents [56]. Research on how changes in co-occurring behaviours are interrelated is warranted in order to further understand possible coaction effects, e.g. how changes in one treated behaviour increase a person’s odds of changing a second treated behaviour [57]. This may be especially needed in the context of the synergistic relationship between alcohol use and tobacco smoking.

## Strengths and Limitations

Strengths of this study include follow-ups over two years, which allowed to investigate both, the comparative efficacy of two delivery channels of BAI as well as trajectories of change in tobacco smoking in the long-term, as previously recommended [17, 19]. Secondly, 81% of all eligible patients participated in our study. This was realized through proactive recruitment. Each general hospital patient was approached and those with at-risk alcohol use offered participation in the intervention, including those with low motivation to change and low alcohol use severity. Satisfactory reach of target populations is warranted for a successful impact of interventions on public health [58]. Thirdly, latent growth analyses were used in order to handle interindividual differences in trajectories of change and missing data appropriately. Using maximum likelihood estimation allowed the inclusion of all participants into the analyses regardless of follow-up participation.

Two study limitations should be considered when interpreting our findings. First, given a lack of consensus about an optimal assessment for tobacco smoking outcome measures [59], and the non-applicability of extensive self-report and biochemical testing as commonly used (e.g. 60) in this study, this study included very brief tobacco outcome measures for various reasons. We wanted to ensure practicability and the participation of a large number of smoking participants as part of our intervention study in a health care setting by keeping the assessment short. Also, we wanted to reduce the burden of participation and therefore enhance the suitability of the intervention for large proportions of smoking participants. This is one of the key factors for interventions in order to have public health impact. Second, as analyses were based on self-reports only, alcohol use and tobacco smoking may be underreported by study participants. However, validity of self-reports on alcohol use and tobacco smoking have not only been shown to be acceptable [61, 62] but are also the foundation of any behavioural intervention.

## Conclusions

This study found that computer-based brief interventions targeting at reducing alcohol consumption may have a positive short-term effect on reduced smoking in at-risk alcohol using participants who also smoke tobacco. Research studies are needed that further explore such additional effects of brief interventions that have rather been neglected in the past. To ensure long-term effects on both behaviours, however, targeting tobacco smoking along with at-risk alcohol use may be more successful.

## Data Availability

The datasets generated and analysed during the current study are not publicly available due to the German data protection law but are available from the corresponding author on reasonable request. Requests will be reviewed for reasonability and compliance with the study purpose and the participants’ informed consent.

## Abbreviations

BAI: Brief alcohol intervention
PECO: “Testing delivery channels of individualized motivationally tailored alcohol interventions among general hospital patients: in person versus computer-based” (Title)
AUDIT: Alcohol use disorder identification test
CONSORT: Consolidated standards of reporting trials
OR: Odds ratios
SD: Standard deviation
*n*: Number of cases
*p*: P-value
CI: Confidence interval
ΔM: Mean net change
