# Correction: Do in-person and computer-based brief alcohol interventions reduce tobacco smoking among general hospital patients? Secondary outcomes from a randomized controlled trial

**DOI:** 10.1186/s13722-023-00430-w

**Published:** 2023-12-20

**Authors:** Filipa Krolo-Wicovsky, Sophie Baumann, Anika Tiede, Gallus Bischof, Ulrich John, Beate Gaertner, Jennis Freyer-Adam

**Affiliations:** 1 Institute for Medical Psychology, University Medicine Greifswald, Walther Rathenau Str. 48, 17475 Greifswald, Germany; 2 German Centre for Cardiovascular Research, Partner Site Greifswald, Fleischmannstr. 42-44, 17475 Greifswald, Germany; 3 Department of Methods in Community Medicine, Institute of Community Medicine, University Medicine Greifswald, Walther Rathenau Str. 48, 17475 Greifswald, Germany; 4 Department of Psychiatry and Psychotherapy, University of Lübeck, Ratzeburger Allee 160, 23538 Lübeck, Germany; 5 Department of Prevention Research and Social Medicine, Institute of Community Medicine, University Medicine Greifswald, Greifswald, Germany; 6 Department of Epidemiology and Health Monitoring, Robert Koch Institute Berlin, Berlin, General Pape Str. 62-66, 12101 Berlin, Germany

**Correction: Addiction Science & Clinical Practice (2023) 18:68** 10.1186/s13722-023-00425-7

After publication of this article [1], it was brought to our attention that the first name and family name of all authors need to be exchanged, the correct author names should be:

Filipa Krolo-Wicovsky, Sophie Baumann, Anika Tiede, Gallus Bischof, Ulrich John, Beate Gaertner and Jennis Freyer-Adam.

The original publication [1] has been corrected.

## References

[1] Krolo-Wicovsky F, Baumann S, Tiede A, Bischof G, John U, Gaertner B, Freyer-Adam J (2023). Do in-person and computer-based brief alcohol interventions reduce tobacco smoking among general hospital patients? Secondary outcomes from a randomized controlled trial. Addict Sci Clin Pract.

